# Carbon Nanotube Yarn Microelectrodes Promote High Temporal Measurements of Serotonin Using Fast Scan Cyclic Voltammetry

**DOI:** 10.3390/s20041173

**Published:** 2020-02-20

**Authors:** Alexander Mendoza, Thomas Asrat, Favian Liu, Pauline Wonnenberg, Alexander G. Zestos

**Affiliations:** Department of Chemistry and Center for Behavioral Neuroscience, American University, Washington, DC 20016, USA; am5748a@alumni.american.edu (A.M.); ta5630a@student.american.edu (T.A.); fl9533a@student.american.edu (F.L.); pw7768a@student.american.edu (P.W.)

**Keywords:** carbon nanotube yarn, fast scan cyclic voltammetry, serotonin, electrochemistry, carbon nanotube

## Abstract

Carbon fiber-microelectrodes (CFMEs) have been the standard for neurotransmitter detection for over forty years. However, in recent years, there have been many advances of utilizing alternative nanomaterials for neurotransmitter detection with fast scan cyclic voltammetry (FSCV). Recently, carbon nanotube (CNT) yarns have been developed as the working electrode materials for neurotransmitter sensing capabilities with fast scan cyclic voltammetry. Carbon nanotubes are ideal for neurotransmitter detection because they have higher aspect ratios enabling monoamine adsorption and lower limits of detection, faster electron transfer kinetics, and a resistance to surface fouling. Several methods to modify CFMEs with CNTs have resulted in increases in sensitivity, but have also increased noise and led to irreproducible results. In this study, we utilize commercially available CNT-yarns to make microelectrodes as enhanced neurotransmitter sensors for neurotransmitters such as serotonin. CNT-yarn microelectrodes have significantly higher sensitivities (peak oxidative currents of the cyclic voltammograms) than CFMEs and faster electron transfer kinetics as measured by peak separation (Δ_EP_) values. Moreover, both serotonin and dopamine are adsorption controlled to the surface of the electrode as measured by scan rate and concentration experiments. CNT yarn microelectrodes also resisted surface fouling of serotonin onto the surface of the electrode over thirty minutes and had a wave application frequency independent response to sensitivity at the surface of the electrode.

## 1. Introduction

Neurochemical detection has proven to be important for the understanding and treatment of several diseases, behaviors, and pharmacological drug states. The detection of dopamine is crucial for understanding Parkinson’s disease and drug abuse [1], while selective serotonin reuptake inhibitors (SSRIs) are used as treatments for depression [2]. Neurochemical measurements are also important in studying other disease states such as epilepsy [3,4] and obesity [5]. In order to study neurochemical dynamics in vivo, scientists have used several bioanalytical assays such as microdialysis [6], positron emission tomography (PET) imaging [7], enzymatic biosensors [8], and carbon electrodes [9]. In comparison to the other techniques, carbon electrodes are biocompatible, have relatively high spatiotemporal resolution [10], are minimally invasive [11], and do not elicit an immune response after implantation. However, carbon fiber-microelectrodes (CFMEs) do have certain drawbacks. CFMEs have been known to have relatively low sensitivities, temporal resolutions, and are susceptible to analyte fouling at the surface of the electrode. To address these issues, novel carbon nanomaterials have been utilized as alternative electrode materials for enhanced neurochemical detection.

Carbon nanotubes, discovered by Iijima and colleagues, were formed by arc discharge synthesis and have served many applications because of their superior mechanical and electrical properties due to high electron delocalization [12,13]. Functionalized carbon nanotube modified microelectrodes were shown to enhance dopamine detection because of the electrostatic interactions of negatively charged end groups with positively charged neurochemicals [14]. Moreover, iron chloride functionalized Carbon Nanotube (CNT) “forest” microelectrodes that displayed vertically aligned CNTs modifying the surface of a carbon fiber-microelectrode. This increased both conductivity and sensitivity at the surface of the electrode [15]. CNT-polymer [16] modified microelectrodes such as Nafion and overoxidized-polypyrrole enhanced the sensitivity dopamine detection and reduced selectivity for anionic ascorbic acid by increasing the negative charge of the surface of the electrode, which causes the electrostatic repulsion of the analytes [17]. Furthermore, gold nanoparticles [18], carbon nanospikes [19], and carbon nanotubes [20] were grown on highly conductive metals to create novel sensors for neurotransmitter detection with fast scan cyclic voltammetry. Despite the great increases in sensitivity afforded by the CNT-modified microelectrodes, there was also a great increase in noise of the measurements because of the heterogeneous surface of the CNT and carbon fiber (loose sheets of graphene) interface. To overcome this issue, novel electrode materials were sought to be constructed solely from CNT sources, not simply CNT-modified carbon fiber electrodes.

Recently, CNT fiber and CNT yarn microelectrodes have been developed as electrode materials for neurotransmitter detection. CNT fibers can be wetspun with the use of surfactants and polymers such as poly(vinyl alcohol) (PVA) [21] and polyethyleneimine (PEI) [22,23]. PVA-CNT fiber microelectrodes were shown to be efficient sensors for biomolecules [11] and showed a resistance toward dopamine fouling [24], but had relatively poor sensitivities [10]. PEI-CNT fiber microelectrodes [25,26] were significantly more conductive than PVA-CNT fiber microelectrodes due to the physisorption of the lone pair of electrons from the amine group to the surface of the CNT fiber, which increased conductivity and enhanced serotonin adsorption to the surface of the electrode. Commercial CNT yarns were utilized as microelectrodes to detect dopamine with fast scan cyclic voltammetry and were found to have a response toward dopamine that was independent of the wave application frequency [27]. It was shown that the increased surface roughness allows for the trapping of dopamine at the surface of the electrode, which prevents desorption of dopamine from the surface of the electrode [28,29]. Comparisons of CNT fiber microelectrodes to CNT yarn microelectrodes also exhibited this property, which illustrates that it is a function of the CNT materials [30]. CNTs were also synthesized via chemical vapor deposition and spun into yarns for the enhanced detection of dopamine in rat brain tissue [31]. Moreover, mechanistic studies were also performed with CNT yarn microelectrodes where defect sites were found to promote the anti-fouling properties of CNT-yarn microelectrodes for serotonin detection with fast scan cyclic voltammetry [32].

Here, we illustrate the use of CNT yarn microelectrodes as enhanced sensors for serotonin and other biomolecules with fast scan cyclic voltammetry. CNT yarn microelectrodes had enhanced sensitivity for serotonin, dopamine, uric acid, ascorbic acids, and other biomolecules. Moreover, they had significantly reduced peak separation (Δ_EP_) than CFMEs, illustrating faster electron transfer kinetics at the surface of a more conductive electrode material. The cyclic voltammograms of dopamine and serotonin were also more easily distinguishable at the surface of CNT yarn microelectrodes than carbon fiber microelectrodes through artifacts of the cyclic voltammograms such as the shape and position (voltage) of their respective reduction peaks. Furthermore, the peak oxidative current for serotonin did not decrease upon repeated injections at the surface of the electrode, thus illustrating the anti-fouling properties at the surface of the electrode. Also, the peak oxidative current for the CVs of serotonin, did not decrease upon increasing the wave application frequency indicating that serotonin was trapped at the surface of the electrode with higher surface roughness. All of this illustrates that CNT yarn microelectrodes serve as enhanced electrode materials for neurotransmitter detection.

## 2. Materials and Methods

### 2.1. Materials

Dopamine, serotonin, ascorbic acid, and uric acid (Sigma-Aldrich, Milwaukee, WI, USA) were used as received. Each 10 mM stock solution was prepared in 0.1 M perchloric acid and diluted with artificial cerebral spinal fluid buffer (aCSF; 145 mM NaCl, 2.68 mM KCl, 1.4 mM CaCl_2_, 1.01 mM MgSO_4_, 1.55 mM Na_2_HPO_4_, and 0.45 mM NaH_2_PO_4_ with pH adjusted to 7.4). Epon 828 Epoxy was obtained from Miller-Stephenson and diethylenetriamine hardener was obtained from Sigma Aldrich. All aqueous solutions were made with deionized water (Millipore, Billerica, MA, USA).

### 2.2. Methods

Carbon fibers (7 µm, Goodfellow, Huntingdon, England) or CNT Yarns (25 µm, obtained from Dr. Vesselin Shanov, University of Cincinnati, Cincinnati, OH, USA) were aspirated into cylindrical glass capillaries (1.2 mm by 0.68 mm, A-M Systems, Inc., Carlsborg, WA, USA) using a vacuum pump (DOA-P704-AA, GAST, Benton Harbor, MI, USA) to prepare CFMEs. The obtained CFMEs were pulled to form two electrodes on a vertical pipette puller (Narishige, model PC-100 and PE-22, Tokyo, Japan), followed by cutting the fiber to lengths of approximately 100–150 microns. CNT yarns were polished at a 90^°^ angle for 30 min with a BV-10 Microelectrode Beveler (Sutter Instruments, San Diego, CA, USA) for an approximate geometric surface area of 5 × 10^−10^ m^2^.

**Scheme 1:**
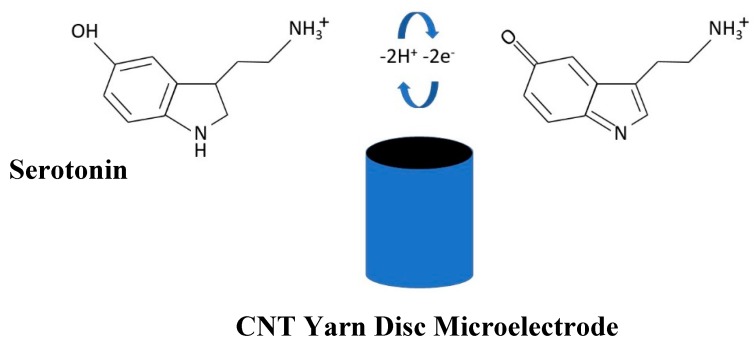
Mechanism of Serotonin Oxidation on a Carbon Nanotube (CNT) Yarn Microelectrode.

Then, protruding carbon fiber or CNT yarn microelectrode tips were dipped in the epoxy hardener mixture (Epon 828 epoxy (Miller-Stephenson, Morton Grove, IL, USA) and diethylenetriamine (Sigma Aldrich), 0.8% by mass resin) for approximately 15 s and then rinsed in acetone to wash away any excess residual epoxy. The electrodes were cured in the oven for 4 h at 125 °C. The mechanism of serotonin oxidation on the surface of a polished CNT yarn microelectrode is shown in Scheme 1.

Fast scan cyclic voltammetry (FSCV) was performed with the WaveNeuro FSCV system with a 5 MΩ headstage (Pine Instruments, Durham, NC, USA). Data were collected using High Definition Cyclic Voltammetry (HDCV) software (University of North Carolina Chapel Hill, Mark Wightman) and a computer interface board (National Instruments PC1e-6363, Austin, TX, USA). A triangle waveform was applied to the electrode from a holding potential of −0.4 V to 1.3 V and back at a scan rate of 400 V/s and a frequency of 10 Hz. The gain of the amplifier was 200 nA/V. A silver–silver chloride wire was used as the reference electrode. Samples were tested in a flow injection analysis system (In Vitro/FSCV Microelectrode Flow Cell with xyz micromanipulator Translational Stage, Pine Instruments, Durham, NC, USA). Buffer and samples were pumped through the flow cell at 1 mL/min using the NE-300 Just Infusion™ Syringe Pump (New Era Pump Systems, Farmingdale, NY, USA). For the traditional waveform, the electrode was scanned from −0.4 to 1.3 V vs. silver–silver chloride (Ag/AgCl, 0.197 V) reference electrode and back at a scan rate of 400 V/s and a wave application frequency of 10 Hz. Electrodes were allowed to equilibrate for approximately 10 min to allow the CFMEs or CNT yarn microelectrodes to equilibrate at the waveform applied and prevent electrode drift between each run. All data was background subtracted to remove any non-faradaic currents by averaging 10 CVs. Electrodes were tested at a flow rate of 1 mL/min using the aforementioned syringe pump.

Scanning electron microscopy images (SEM) images were obtained with a JEOL JSM-IT100 (JEOL, Tokyo, Japan). CNT yarn microelectrodes were gold sputtered to prevent charging. The working distance was set to 10 mm and the accelerating voltage was 10 kV. Energy-dispersive X-ray spectroscopy (EDS/EDX) measurements was also performed to identify chemical compositions of the CNT yarn microelectrodes.

All data analysis was performed by using Graph Pad Prism 7. Statistical analysis was performed with a student’s t-test. Statistical significance was set to *p* < 0.5. All error bars are standard error of the mean (SEM) unless otherwise noted.

## 3. Results

The optical and chemical characterization of CNT-yarn microelectrodes was performed with scanning electron microscopy (SEM) imaging and EDS/EDX characterization using a JEOL JSM-IT100 electron microscope. Before the CNT yarn could be utilized as an electrode for the electrochemical sensing of neurotransmitters with fast scan cyclic voltammetry, it has to be characterized optically to examine the surface features to determine whether it was suitable for neurotransmitter adsorption. Most of the CNT yarns are either formed from liquid-state spinning and solid-sate spinning as previously described [33,34]. Synthetic fibers are formed from a concentrated, viscous liquid. However, in liquid-based spinning, CNTs are dispersed into fluids and either extruded or coagulation spun into fibers. In either process, long vertical arrays of CNT-yarns are formed from individual fibers of “fibrils” twisted together to form CNT yarns.

SEM imaging of CNT yarns reveals fine surface features that appear efficacious for neurotransmitter sensing measurements. At a relatively low magnification (×250), we show an entire CNT yarn. As opposed to the carbon fiber microelectrode, the CNT yarn microelectrode is approximately three times as large (25 microns in diameter) in comparison to the carbon fiber microelectrode (7 microns in diameter). This is primarily important because the surface of the microelectrode surface is directly proportional to the sensitivity Randles-Sevcik equation for voltammetry experiments. In other words, larger electrodes with higher surface areas will be able to detect lower concentrations (lower limits of detection) of biomolecules. This is important as biomolecules are usually found in low (sub-micromolar and nanomolar concentrations) levels in biological tissue and other samples.

**Figure 1:**
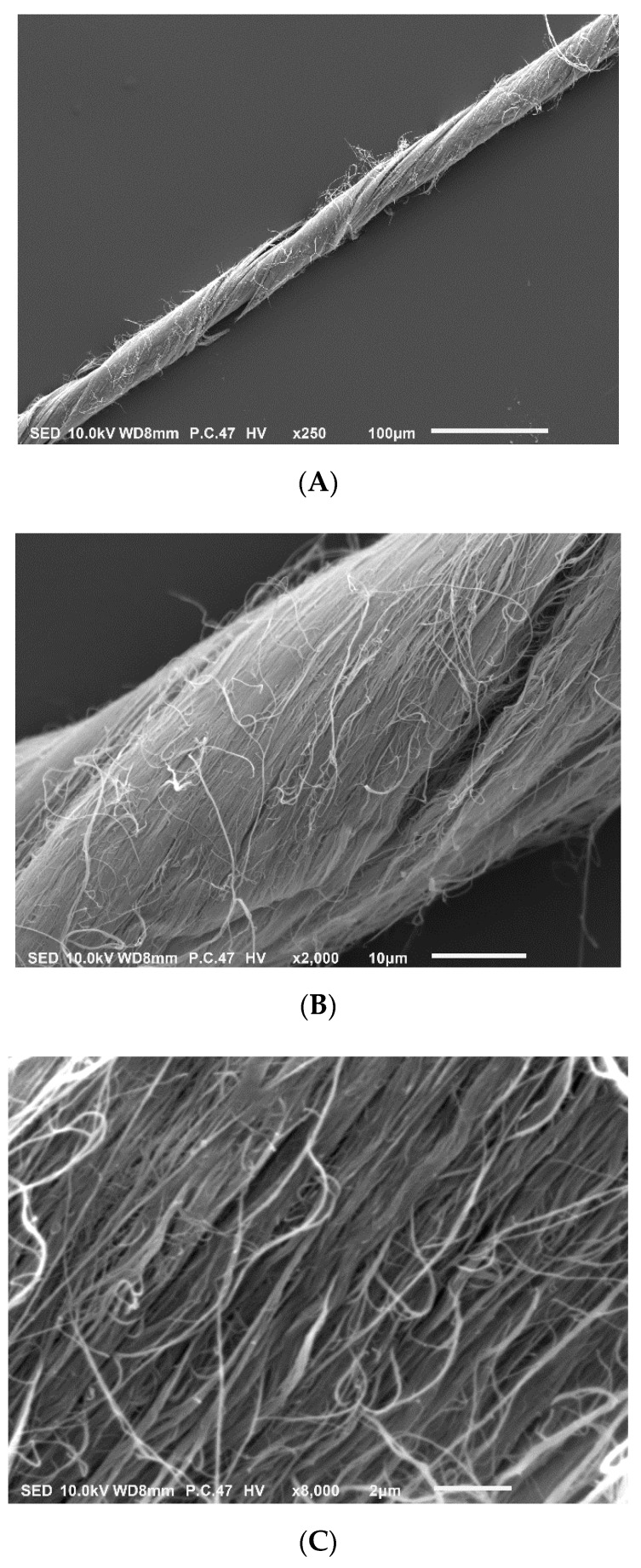
Scanning electron microscope (SEM) images of CNT yarn fibers at various zoomed-in magnitudes displaying (**A**) zoomed-out magnification display (×250) of microelectrode. (**B**) Side of CNT yarn microelectrode and presence of individual fibrils twisted to form the CNT yarn surface (×2000 magnification). (**C**) Cross-section of CNT yarn microelectrode and presence of individual fibrils twisted to form the CNT yarn surface (×6000 magnification).

In Figure 1B,C, we show the zoomed-in magnifications of the CNT yarn microelectrodes at ×2000 and ×6000, respectively. The surface features of the CNT yarn microelectrodes are significantly different than the CFMEs. First and foremost, the carbon fibers are primarily smooth with only mild indentations (data not shown). However, the CNT yarn microelectrodes show the individual wrapping of tiny fibrils that are woven into yarns. It is hypothesized that these fibers or fibrils (approximately 50 nm in diameter) are actually individual bundles of CNTs drawn out through an extrusion process and wet spinning with the help of a graphite furnace. These fibrils are then twisted individually together to form CNT yarns. The aspect (surface to volume) ratio and surface roughness is also efficacious for neurotransmitter detection. The more pronounced surface features promote neurotransmitter adsorption to the surface of the microelectrode, thus enhancing the neurochemical detection at the surface of the microelectrode. Also, CNT yarn microelectrodes have a higher concentration of edge-plane carbon, which is the catalytic site for neurotransmitter adsorption as opposed to basal plane of carbon [35].

**Figure 2:**
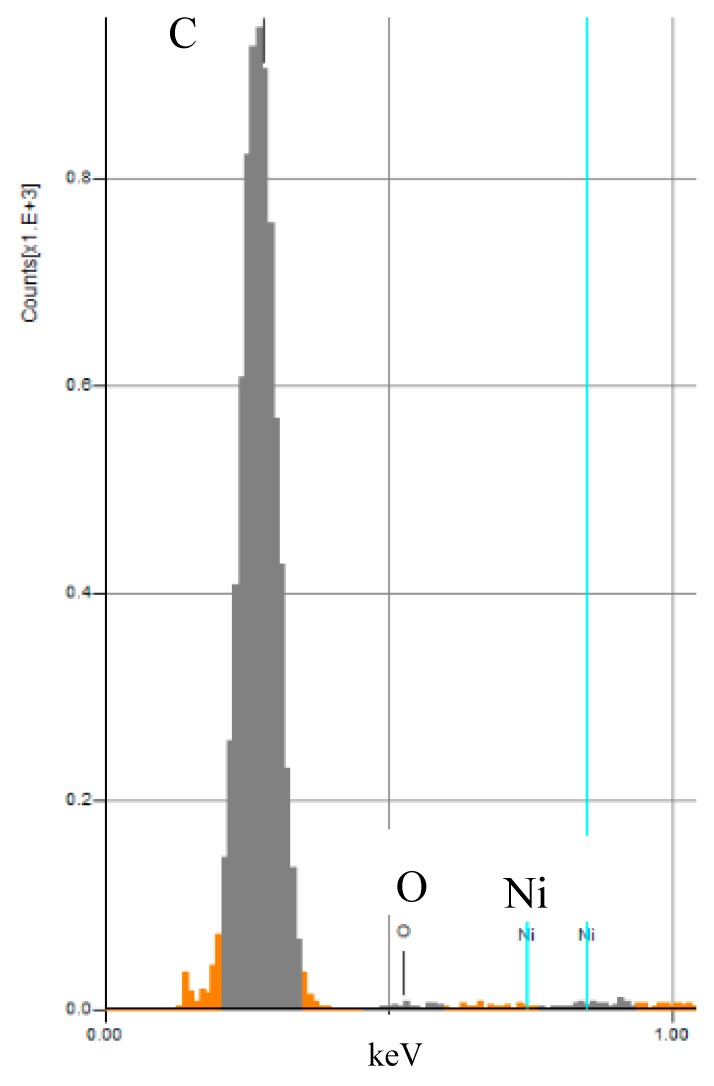
Energy-dispersive X-ray spectroscopy (EDS/EDX) measurements of chemical functionalities at the surface of the CNT yarn microelectrodes. The most abundant elements present are carbon, oxygen, and nickel.

In Figure 2, below, we show the surface chemical functionalization of CNT yarn microelectrodes. As expected, the most prevalent element is carbon as CNTs are formed primarily from carbon usually derived from acetylene gas during the chemical vapor deposition process. Also, there were trace amounts of surface functionalities present such as oxygen and nickel. Primarily, nickel is utilized as a catalyst for the growth of vertically aligned CNTs, which could be the reason for the presence of trace amounts of this heavy metal [36,37]. Moreover, the presence of oxygen could explain the oxidation of carbon-carbon bonds either in air or electrochemically. The presence of oxygen is fundamentally important in creating a novel neurotransmitter sensor. Carbon modified with negatively charged oxide, hydroxy, ketone, carboxylic acid, and other moieties makes the electrode more negatively charged. Therefore, the electrodes are more sensitive to positively charged catecholamines and monoamines such as dopamine and serotonin for example. The monoamines are protonated at a physiological pH of 7.4, which makes them positively charged and enables them to adsorb onto the surface of the negatively charged microelectrode surfaces through an electrostatic attraction of opposite charges, which enhances neurotransmitter detection [38,39].

### 3.1. Electrochemical Measurements

The CNT yarn microelectrodes were then utilized as electrochemical sensors for the detection of several biomolecules. The neurotransmitters that we chose were the catecholamine dopamine, the monoamine serotonin, and the anionic interferants ascorbic acid, and uric acid. Dopamine is an important catecholamine and monoamine neurotransmitter important for understanding the Parkinson’s Disease [40], amphetamine abuse [41,42], cocaine abuse [1], and other decision-making capabilities. Serotonin regulates mood and behavior and the depletion of serotonin in the synaptic cleft causes depression [43]. The administration of SSRIs such as escitalopram helps treat depression by increasing serotonin levels in the synaptic cleft [44,45,46]. Therefore, the detection of these neurotransmitters with fast scan cyclic voltammetry and CNT-yarn microelectrodes will further help understand their physiological roles.

**Figure 3:**
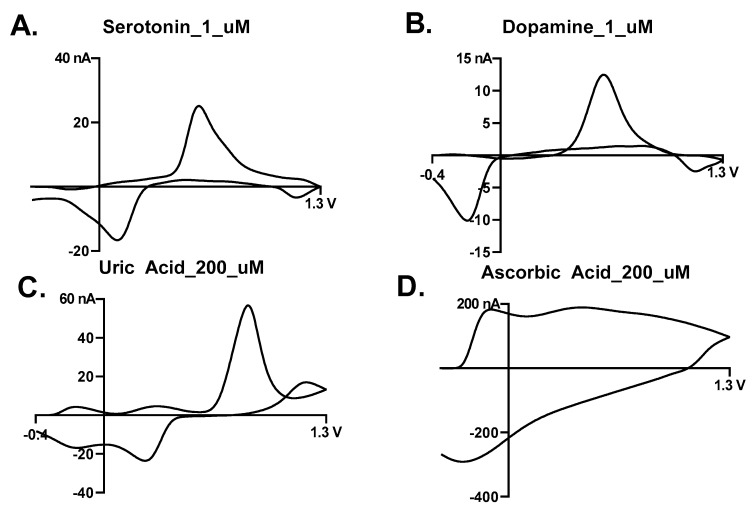
Cyclic voltammograms (CVs) of biomolecular neurotransmitters and other analytes using fast scan cyclic voltammetry (FSCV) with CNT-yarn microelectrodes. The CNT yarn microelectrodes were able to detect (**A**) 1 μM serotonin, (**B**) 1 μM dopamine, (**C**) 200 μM uric acid, and (**D**) 200 μM ascorbic acid.

In Figure 3 we show the cyclic voltammograms of (A) 1 μM serotonin, (B) 1 μM dopamine, (C) 200 μM uric acid, and (D) 200 μM ascorbic acid. The monoamines (serotonin and dopamine) have higher sensitivities at the surface of the electrode because the amines are protonated, and hence positively charged, and thus, adsorb to the negatively charged electrode surface through the electrostatic interaction of opposite charges. The sharp peak shaped currents denote adsorption of the monoamines to the surface of the electrode as opposed to diffusion control. CNT yarn microelectrodes are also more conductive than the conventional graphitic CFMEs, thus explaining the faster electron transfer and faster Δ_EP_ (peak separation) with respect to CFMEs. The electrodes are also larger, explaining the increased sensitivity in comparison to CFMEs.

On the other hand, negatively charged biomolecules (C) uric acid and (D) ascorbic acid have to be tested at significantly higher concentrations in order to be detected by the CNT-yarn microelectrode. It is hypothesized that they are diffusion controlled to the surface of the negatively charged CNT-yarn microelectrode that is functionalized with negatively charged oxide groups. This creates an electrostatic repulsion at the surface of the electrode with the anionic analytes, which prevents adsorption at the surface of the CNT yarn microelectrode, hence the lower sensitivity.

### 3.2. Adsorption Control

**Figure 4:**
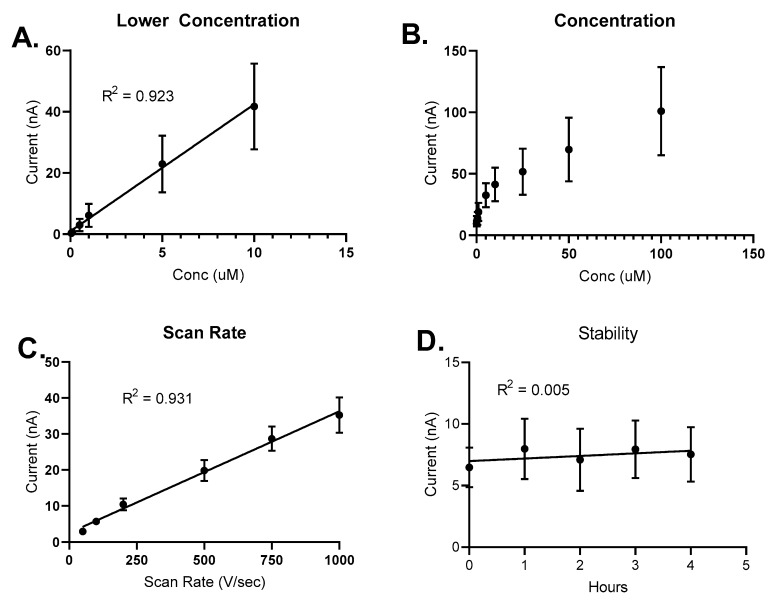
Adsorption Properties. For CNT yarn microelectrodes. (**A**) The linear range for serotonin measurements is from 100 nM to 10 μM, *n* = 4. (**B**) Serotonin is saturated at the surface of the electrode after 10 μM, which is shown by the asymptotic curve and deviation from linearity. (**C**) Peak oxidative current for serotonin CVs were linear with respect to scan rate. (**D**) The peak oxidative current response with respect to serotonin was stable over four hours.

We then performed a series of FSCV experiments on CNT yarns to examine the adsorptive properties of serotonin to the surface of the CNT-yarn microelectrode. First, we varied the concentration of serotonin from 100 nM to 100 μM. As expected we saw a linear relationship with respect to the peak oxidative current (Figure 4A, *n* = 4, R^2^ = 0.923). At concentrations higher than 10 μM, serotonin became saturated at the surface of the electrode, hence producing the asymptotic curve and deviation from linearity (Figure 4B), which denoted more diffusion controlled at the surface of the electrode.

We further altered the scan rate from 50 V/sec to 1000 V/sec. We also observed a linear relationship between peak oxidative current and scan rate (Figure 4C, *n* = 4, R^2^ = 0.931) indicating adsorption control at the surface of the electrode. Moreover, we performed a stability experiment where we injected 1 μM serotonin repeatedly onto the surface of the CNT yarn microelectrode once per hour for a total of four hours. There was no significant difference in the peak oxidative current for serotonin detection over four hours (Figure 4D) indicating a stability of the CNT yarn microelectrode response over a four-hour time period.

### 3.3. Anti-Fouling Properties

Moreover, we also studied the anti-fouling properties of the CNT-yarn microelectrodes. At the surface of the CFMEs, serotonin is known to polymerize (or foul) the surface of the electrode by becoming a dimer and then undergoing a free-radical polymerization at the surface of the electrode. The subsequent polymer creates a non-conductive coating on the surface of the CFME, which blocks the further adsorption and electron transfer of serotonin and other biomolecules to the surface. Also, the breakdown metabolite product of serotonin, 5-hydroxyindoleacetic acid (5-HIAA), also fouls the surface of electrode like serotonin and is found in vivo at concentrations almost 10× higher than serotonin in certain brain regions.

Usually, it is necessary to electrodeposit a negatively charge ion exchange polymer such as Nafion to the surface of the electrode to make it negatively charged to electrostatically repel the anionic 5-HIAA from the surface of the electrode. However, this decreases the temporal resolution and, hence, increases the response time for serotonin measurements by creating an extra polymer layer that the analyte must diffuse through before reaching the electrode surface. Therefore, other electrode materials must be utilized to enhance the serotonin detection such as CNT yarn microelectrodes.

**Figure 5:**
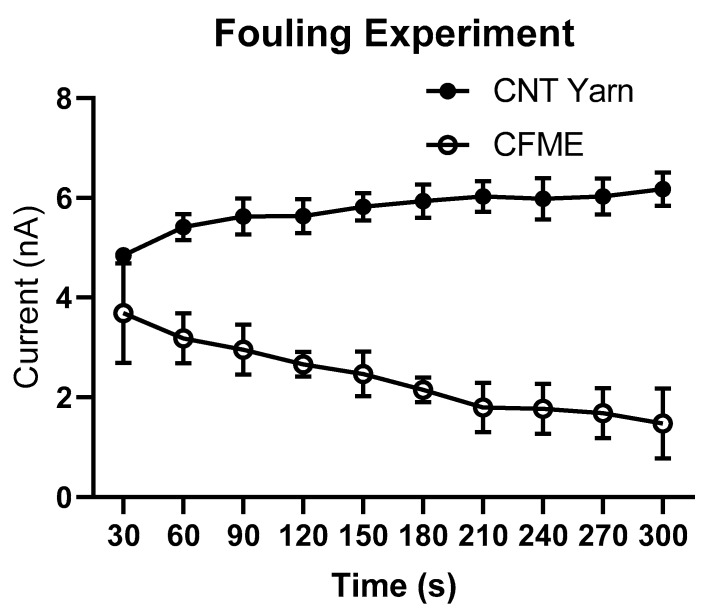
Fouling experiment. Increasing the injection number decreases the peak oxidative current for serotonin detection by over 50% for carbon fiber-microelectrodes (CFMEs), but remains unchanged for CNT yarn microelectrodes, *n* = 5.

We compared CNT yarn microelectrodes to CFMEs for their anti-fouling properties with respect to repeated instantaneous injections of 1 μM serotonin for over five minutes or approximately 10 injections (approximately 30 s per run). As shown in Figure 5, for CFMEs, there was an over 50% decrease in peak oxidative current of the cyclic voltammograms for 1 μM serotonin detection between the first and the tenth run. However, there was no marked decrease in the sensitivity for CNT-yarn microelectrodes that underwent the same experiment. We hypothesize that serotonin fouled the surface of the CFMEs, but not the surface of the CNT yarn microelectrodes. The most likely cause for this phenomenon was the presence of more edge plane carbon (sp^3^ hybridized at the surface), which is the catalytic site for neurotransmitter oxidation, and defect sites at the surface of the CNT yarn microelectrodes, which prevented surface fouling on CNT yarn microelectrodes [32,35,47].

### 3.4. High Temporal Measurements (Wave Application Frequency Independence)

**Figure 6:**
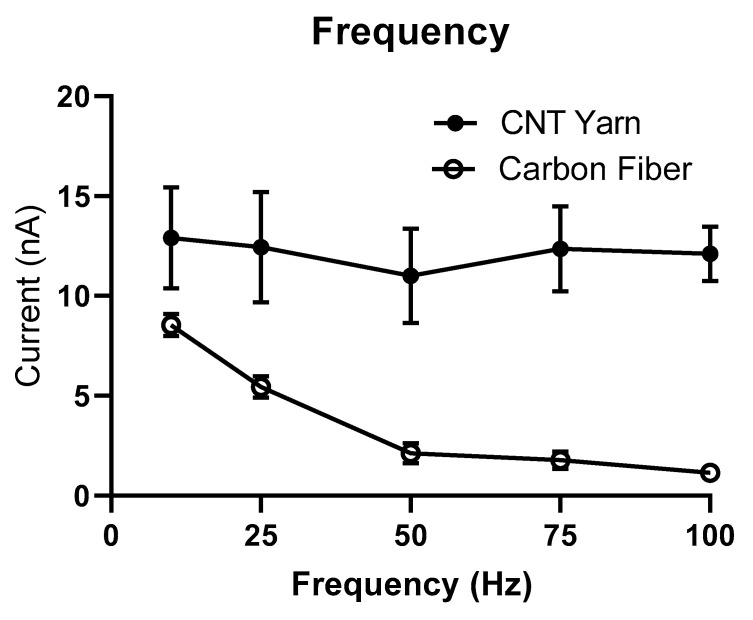
Frequency experiment. Increasing the wave application frequency decreases the peak oxidative current for serotonin detection on CFMEs, but not on CNT yarn microelectrodes, *n* = 5.

Lastly, we have shown that CNT yarn microelectrodes have a frequency independent response with respect to serotonin detection (Figure 6). Usually, increasing the wave application frequency (i.e., from 10 Hz to 100 Hz) lowers the amount of time at the negative holding potential, which decreases the time for adsorption onto the electrode surface and, hence, decreases sensitivity as well. Previous studies have shown that the peak oxidative current on the cyclic voltammograms of dopamine detection for CNT yarn microelectrodes was independent of the wave application frequency. However, up until now, this has not been studied with other monoamines such as serotonin. The rationale is that CNT yarn microelectrodes are more sp^2^ hybridized than carbon fiber electrodes, which are primarily composed of loosely ordered sheets of that are partially sp^2^ and sp^3^ hybridized. Therefore, the mechanism of adsorption onto CFMEs for dopamine or serotonin is primarily thought to be charge-related. In other words, the protonated amine of dopamine or serotonin causes it to be positively charged, which is electrostatically attracted to and, hence adsorbs, onto the negatively charged oxide groups on surface of the surface of the CFME. However, another mechanism of adsorption may occur onto the more sp^2^ hybridized CNT yarn microelectrode including π–π stacking from the phenyl group of the catechol dopamine to the surface of the carbon electrode in addition to hydrogen-bonding from catechol group to the oxide functionalized electrode surface as well.

Recently, the Venton group has shown that CNT-yarn and fiber microelectrodes have higher surface roughness than CFMEs and have been shown to trap dopamine in micro-crevices that prevents desorption from the surface, which can explain the peak oxidative current independence from frequency [28,29]. As shown in this work, increasing the wave application frequency from 10 Hz to 100 Hz decreases the peak oxidative current for serotonin detection on CFMEs by over 90%. However, increasing the wave application frequency from 10 Hz to 100 Hz has no significant effect on the sensitivity (peak oxidative current) for serotonin detection onto CNT-yarn microelectrodes. If anything, the peak oxidative current slightly increases as the electrode oxidizes and equilibrates when scanning at higher wave application frequencies. We hypothesize that, like dopamine, serotonin is trapped at the surface of the micro-crevices of the increased surface roughness of the CNT yarn microelectrode, which causes the serotonin to be stuck at the surface of the CNT yarn electrode and prevents desorption, thus explaining this phenomenon. As far as we know, this is the first study that has shown the wave application frequency independence for serotonin detection on CNT yarn microelectrodes.

## 4. Conclusions and Future Work

In this study, we have shown a novel application for CNT-yarn microelectrodes and further characterization for serotonin detection. CNT yarn microelectrodes had high surface roughness and were functionalized with negatively charged oxide groups, which made them ideal for electrode materials for neurotransmitter detection. They also had higher sensitivities toward cationic monoamines dopamine and serotonin with respect to anionic interferants. CNT-yarn microelectrodes displayed an adsorption control dependence with respect to serotonin oxidation detection in addition to anti-fouling properties in comparison to traditional CFMEs. Moreover, high temporal measurements for serotonin were illustrated for the first time as CNT yarn microelectrodes displayed a frequency independent response with respect to serotonin. This work could allow for the improved measurement of serotonin at high temporal resolution in biological tissue, which could help enhance the understanding of depression and other disease states through the rapid measurements of serotonin.
